# Disseminated Histoplasmosis With Lung and Liver Involvement in an Immunocompetent Patient

**DOI:** 10.7759/cureus.75228

**Published:** 2024-12-06

**Authors:** Osvaldo Alexis Marché Fernández, Luis José Pinto García, Jesús Guillermo Hernández García, César Daniel Alonso Bello, Juan Daniel Díaz García

**Affiliations:** 1 Internal Medicine, Hospital Juarez de Mexico, Mexico City, MEX; 2 Nephrology, Hospital Clinic Barcelona, Barcelona, ESP

**Keywords:** fungal infection in immunocompetent, fungal lung infection, fungal pneumonia, invasive fungal infections, tropical infections

## Abstract

Histoplasmosis, caused by the fungus *Histoplasma capsulatum*, is a significant public health concern in endemic regions like Mexico. Immunocompromised individuals, especially those with HIV infection and those exposed to nitrogen-rich environments, such as bird excrement or bat guano, are particularly vulnerable. This case report describes a middle-aged patient with jaundice in the skin and mucous membranes. Suspecting cholecystitis, abdominal imaging was performed, revealing hepatomegaly and bilateral pulmonary infiltrates with consolidations and nodular lesions on computed tomography. After being transferred to the internal medicine unit, a comprehensive diagnostic evaluation was carried out. Despite negative results from HIV serology and acid-fast bacillus testing, the patient tested positive for urine *Histoplasma* antigen, confirming the diagnosis of disseminated histoplasmosis. This case underscores the importance of considering histoplasmosis in the differential diagnosis of patients with relevant risk factors presenting with systemic symptoms.

## Introduction

*Histoplasma capsulatum* serves as the primary etiological agent of histoplasmosis, a condition primarily characterized by pulmonary manifestations. Nevertheless, it may also induce alterations in other tissues [1]. Traveling or living in an endemic area represents a major risk factor for histoplasmosis infection [2,3]. Mexico is considered an endemic region for this disease, but there are no updated epidemiological data on its prevalence, or the number of cases reported annually [4]. The most recent data available pertains to the records from the National Institute of Epidemiological Diagnosis and Reference (INDRE), which document a cumulative total of 1444 histoplasmosis cases spanning the period from 1953 to 1997 [5]. In general, this microorganism inhabits environments rich in nitrogen, such as bat guano or bird feces, which can be found in caves [1]. For the disease to manifest, inhalation of minute mycelium fragments and microconidia is imperative, followed by their traversal across the lung epithelium, subsequently being phagocytosed by macrophages and neutrophils [1]. In immunocompetent patients, the disease typically remains asymptomatic in up to 90% of cases [3]; however, in immunodeficient individuals, it is characterized by symptoms such as cough, dyspnea, fever, and malaise [2].

Immunocompromised patients, particularly those undergoing treatment with steroids, immunomodulatory drugs, or those afflicted with primary immunodeficiency, are predisposed to developing extrapulmonary manifestations with greater frequency [6]. Among the prevalent symptoms observed, fever (89.1%), respiratory symptoms (38.1%), weight loss (37.4%), splenomegaly (72%), hepatomegaly (68.1%), and lymphadenopathy (41.2%) are frequently reported) [6,7].

The management of this pathology continues to follow the guidelines proposed by the Infectious Diseases Society of America (IDSA) in 2007 [8]. In cases of disseminated disease, amphotericin B (either liposomal or deoxycholate) should be administered, followed by itraconazole [8]. This disease is common in our environment, making it imperative to establish a culture of prevention along with adequate prophylactic management.

## Case presentation

**Figure 1:**
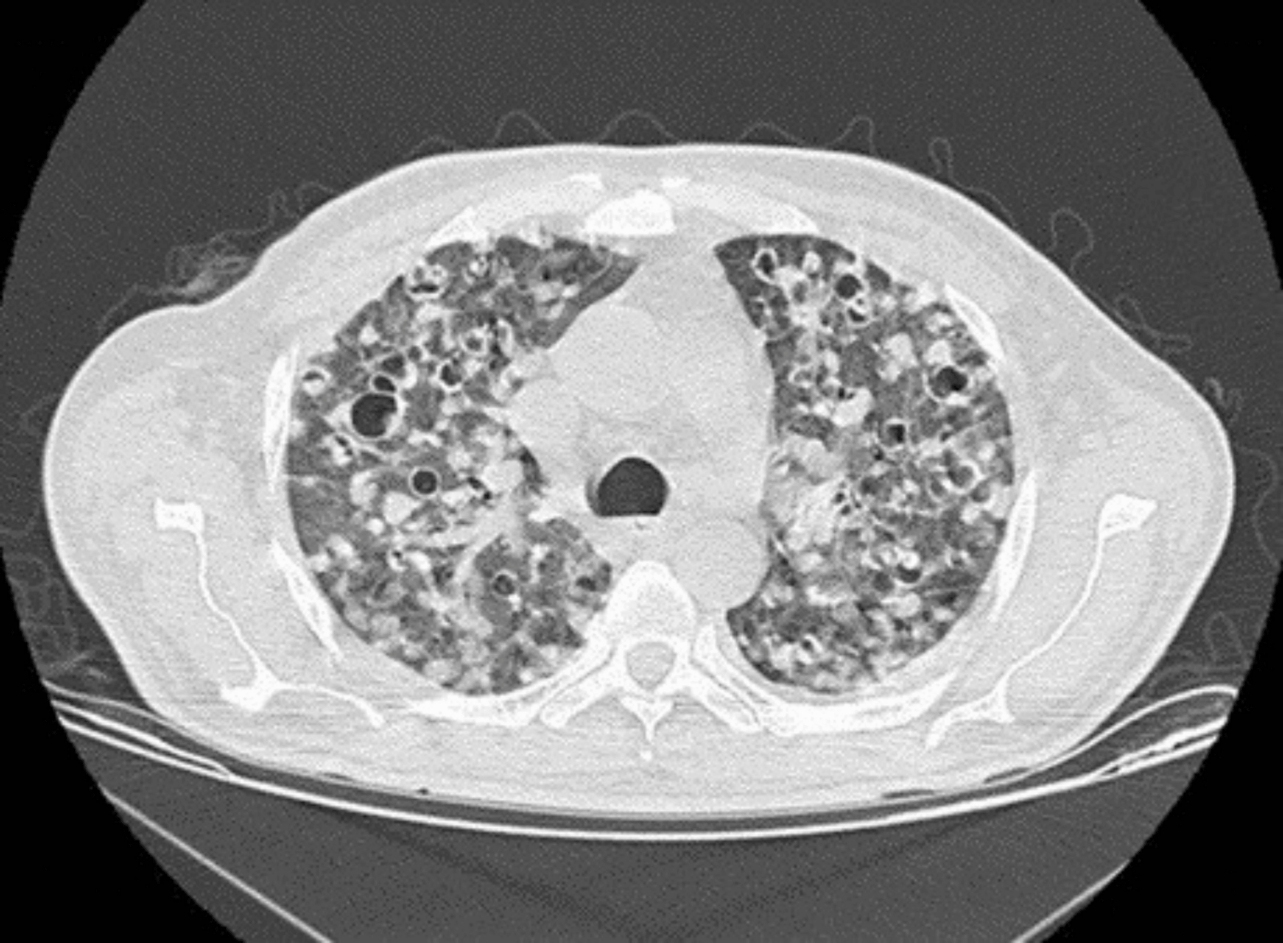
Chest computerized axial tomography in a high-resolution window. A simple computerized axial tomography is presented, in a high-resolution window, in a five-vessel section. At the lung level, multiple hyperdense lesions in a nodular pattern can be seen, to which is added an image of multiple bronchial dilatations.

We present the case of a male patient in his fifties with an unremarkable medical history, except for smoking and occasional alcohol consumption, which consists of drinking 500 ml of beer once a month for three years. He mentioned during the interrogation that he had traveled outside of the Mexico City metropolitan area two months before being admitted to the hospital. He had stayed in a house with poor hygiene conditions where there was no drainage or sewage system. Moreover, the water was not purified or dewormed. The patient was admitted to the emergency department with a clinical picture comprising of fever, unintentional weight loss (approximately 5 kg in a month), dyspnea, respiratory distress, and malaise. Upon physical examination, the patient had the following vital signs: heart rate of 110 beats per minute, respiratory rate of 25 breaths per minute, blood pressure of 130/80 mm Hg, temperature of 37.5°C, and an oxygen saturation of 84% in room air. Upon examination of the thorax, crackling sounds were auscultated in both lung fields, in addition to hypoventilation and decreased expansion and contraction movements. On the other hand, the examination of the abdominal region revealed hepatomegaly, and splenomegaly, with pain upon palpation in the right upper quadrant but not in any other quadrant. Later, edema in both of his pelvic limbs along with jaundice on the skin and mucous membranes (ophthalmic and oral) was added to the symptoms. The patient was admitted to the general surgery department with suspected cholecystitis. Subsequent thoracic and abdominal tomography revealed bilateral pulmonary infiltrates alongside hepatomegaly (25 cm in length) and splenomegaly (15 cm in length). The tomography report detailed areas of consolidation, ground glass opacities, and multiple nodular images in the lungs (Figure 1).

As part of the diagnostic workup, HIV, HCV, and HBV surface antigen serology tests were conducted, and all three yielded negative results. Additionally, a sputum smear test was performed to detect *Mycobacterium tuberculosis*, which also came back negative. The sputum nucleic acid amplification test was also negative. Additionally, serum glucose was requested with a result of 150 mg/dL, leukocytes with 16,000 cells per microliter, an erythrocyte sedimentation rate of 24 mm/h, and liver function tests that showed an aspartate aminotransferase (AST)/alanine aminotransferase (ALT) ratio of 1.79 and an R factor of 0.4 (cholestatic). Upon suspicion of atypical pathogens, a urine *Histoplasma* antigen test was requested and reported as positive, leading to the diagnosis of disseminated histoplasmosis.

**Figure 2:**
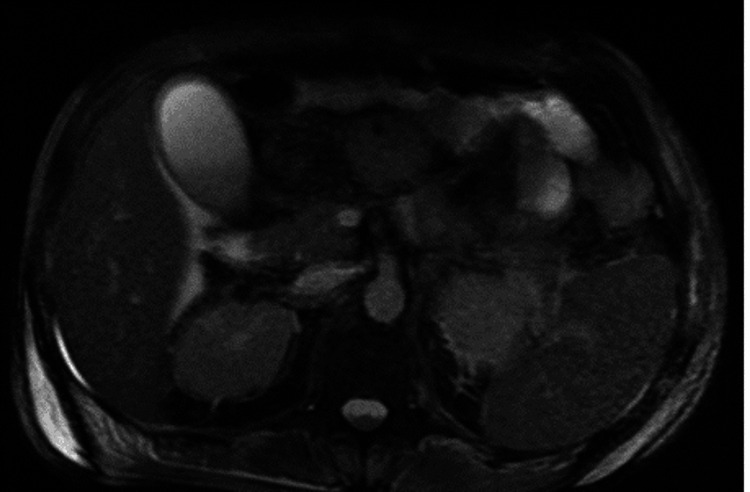
Magnetic resonance cholangiography. Magnetic resonance cholangiography in axial section, in FIESTA sequence. This cut allows us to observe the presence of the gallbladder together with the common and cystic hepatic duct. None of the structures present filling defects in their lumens. FIESTA: fast imaging employing steady-state acquisition.

As part of the diagnostic workup, sputum, and blood cultures were requested, as well as myelocultures to rule out infiltration of *Histoplasma capsulatum* into the bone marrow. The first ones showed discouraging results by not isolating a specific pathogen. On the other hand, in the myelocultures there was no growth of fungal agents, and the Groccot and Wright staining did not identify any microorganisms. Also, a bronchoscopy was performed with bronchial biopsy, finding bronchial parenchyma with chronic and acute inflammation, with little hyaline mucus. Due to the presence of liver damage, we sought to rule out other causes that could cause it. For this reason, a magnetic resonance cholangiography (Figure 2) was performed, in which the absence of structural or obstructive changes in the bile duct was certified.

To rule out the immunocompromise status, sequence analysis and deletion/duplication testing of the 574 genes were performed (Inborn Errors of Immunity and Cytopenias Panel, Invitae, San Francisco, USA).

A variant of uncertain significance, c.4049C>T (p.Thr1350Met), was identified in ATM; this gene is associated with autosomal dominant predisposition to breast, ovarian, pancreatic, and prostate cancer, which is also associated with Ataxia-Telangiectasia. However, this polymorphism does not appear to be causally linked to the disease. Consequently, the individual is considered immunocompetent.

In agreement with the infectious diseases department of our institution, it was decided to administer amphotericin B deoxycholate calculated at 1 mg/kg of weight every 24 hours for a period of two weeks. After one week of treatment, clinically the patient showed a decrease in jaundice, improvement in respiratory sounds in the pulmonary fields, and no abdominal pain. Biochemically, the results showed total bilirubin of 0.90 mg/dL (previously 3.33 mg/dL), direct bilirubin of 0.77 mg/dL (previously 2.86 mg/dL), AST of 60 mg/dL (previously 244 mg/dL), ALT of 75 mg/dL (previously 372 mg/dL), and an erythrocyte sedimentation rate (ESR) of 18 mm/h (previously 24 mm/h).

Subsequently, therapy with itraconazole 200 mg every eight hours for three days was indicated; and, finally, 200 mg every 24 hours until completing one year.

**Figure 3:**
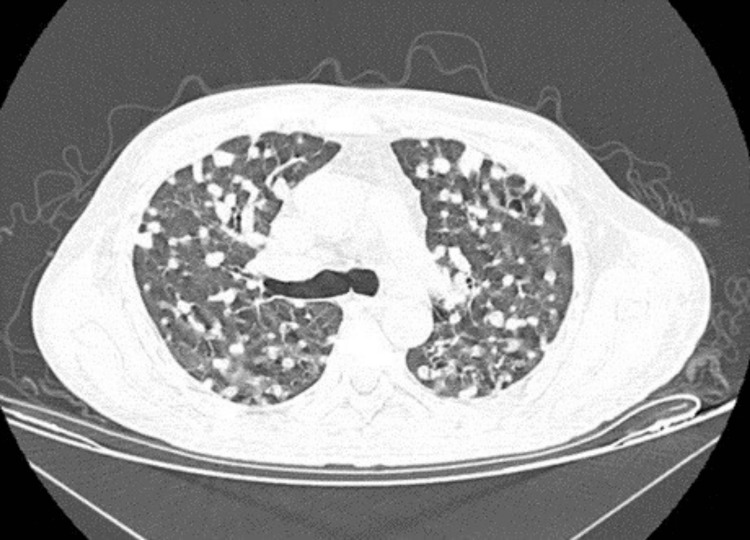
Post treatment chest computerized axial tomography in a high-resolution window. A simple computerized axial tomography is presented, in a high-resolution window, in a five-vessel section. At the lung level, lesions compatible with a nodular pattern can be seen, with improvement compared to a previous study shown in Figure 1.

One month later, a control chest computed tomography was requested (Figure 3). It shows a decrease in the size of the nodular lesions and an improvement in the lung parenchyma. In addition, the patient showed decreased liver function tests but continued to have hepatomegaly in the tomographic study (21 cm in length).

Up to now, the patient has been monitored through the outpatient department of the infectious diseases department. We obtained written informed consent from the patient to use the information from their case.

## Discussion

Histoplasmosis is a disease that presents as an infection in the respiratory tract [1]. In human beings there are only two organisms that can cause this disease, which are *Histoplasma capsulatum* *var dubossii* and *Histoplasma capsulatum var capsulatum*, the first one being more prevalent in the regions of central and western Africa, while the second has a worldwide distribution [3].

Infection in humans occurs when microconidia are inhaled, joining the family of integrins CD11 and CD18, which allows recognition and subsequent phagocytosis by neutrophils and macrophages [1]. After the phagocytosis process, the fungus multiplies exponentially within macrophages [6], later transforming into yeasts and, in this way, migrating to lymph nodes and distant tissues (liver and spleen) [1-6]. In immunocompetent individuals, disseminated infections can be controlled by the adaptive immune system and the secretion of cytokines [6]. However, in patients with some degree of immunosuppression, this infection may progress or reactivate from an old infectious focus, leading to various clinical manifestations [6].

The acute pulmonary infection develops after an incubation period for the fungus of seven to 21 days [1]. This condition is characterized by respiratory symptoms such as cough and dyspnea [1,2], as well as general symptoms including fever, asthenia, weakness, and chills [2]. Chest X-rays typically reveal a pattern of diffuse bilateral pulmonary infiltrates along with mediastinal and hilar adenopathy, without evidence of pleural involvement [1-7]. Although the symptoms are usually self-limited within a period of two weeks, patients exposed to large amounts of the fungal inoculum may develop acute respiratory distress syndrome [1]. On the other hand, the subacute phase occurs weeks to months after infection [2] and is characterized by the presence of constitutional symptoms along with a greater deterioration of pulmonary function [3]. The chronic phase of the disease occurs months to years after infection [2]. Generally, immunosuppressed patients or those with a history of lung disease will develop this condition [1], which is primarily characterized by weight loss, fatigue, chills, severe dyspnea, and chest pain [1]. Chest X-rays often reveal a nodular pattern in both lungs, as well as in the mediastinal and hilar regions, along with pleural thickening, areas of consolidation, and cavitations. Additionally, disseminated histoplasmosis develops more easily, with the liver being a commonly affected organ in up to 90% of cases [6]. Liver abnormalities include jaundice, stigmata of chronic liver disease, portal hypertension, ascites, and esophageal varices. Additionally, there is an elevation of both transaminases. In the study conducted by Spec et al., it was noted that an AST/ALT ratio of 2.5 or higher (95% CI: 1.22-4.16) was more strongly correlated with disseminated histoplasmosis than with other mycoses or pulmonary histoplasmosis, which exhibited a lower AST/ALT ratio (P < 0.0001) [9].

In the case of the patient we present, he was in the subacute phase of the infection, as he exhibited respiratory involvement along with systemic symptoms (weight loss, jaundice, hepatomegaly, and splenomegaly).

The gold standard for diagnosing histoplasmosis is the identification of yeasts in histopathology or cytopathology of clinical samples, as well as the growth of the mold in culture incubated at room temperature [7]. However, due to the limitations of fungal culture, other diagnostic tools have been developed, leading to the use of serological methods [1-3].

A multicenter prospective cohort study conducted by Hage et al. demonstrated that urine *Histoplasma* antigen is an excellent diagnostic tool for histoplasmosis [10]. In this study, 218 patients from eight medical centers diagnosed with histoplasmosis were evaluated; of these, 158 had disseminated histoplasmosis and 60 had pulmonary histoplasmosis (six in the acute phase, 46 in the subacute phase, and eight in the chronic phase). Various diagnostic methods for detecting histoplasmosis were employed for all patients, and the determination of *Histoplasma* antigen in urine was positive in 91.8% of patients with disseminated disease (95% CI, 86.3%-95.1%), 94.6% of patients with AIDS, 93.1% of those with other conditions causing immunosuppression, and 73.3% in immunocompetent individuals (P = 0.837). In addition, urinary antigen concentration was found to be higher in patients with disseminated disease compared to those with only pulmonary involvement (mean 11.32 vs 0.77 ng/mL; P < 0.001). On the other hand, in patients with only pulmonary histoplasmosis, urinary antigen was present in 83.3% of those who were in the acute phase, 30.4% of those who were in the subacute phase, and 87.5% of those who were in the chronic phase. In conclusion, the detection of urinary antigens represents an adequate diagnostic tool for disseminated histoplasmosis [10]. There are also other diagnostic methods like the detection of antibodies and molecular methods that can be useful for the diagnostic approach of histoplasmosis [1].

The differential diagnoses of histoplasmosis can range from infectious causes such as pulmonary tuberculosis, coccidioidomycosis, and aspergillosis, to neoplastic or infiltrative causes such as lung cancer and sarcoidosis [7].

Management of acute histoplasmosis is generally not necessary; however, the Infectious Diseases Society of America (IDSA) guidelines propose itraconazole at a dosage of 200 mg every eight hours for three days, followed by 200 mg every 24 hours for six to 12 weeks [8]. In cases of severe or disseminated histoplasmosis, the initiation of medical therapy with liposomal amphotericin B at a dose of 3 mg/kg (or amphotericin B deoxycholate at 0.7 to 1 mg/kg per day) is recommended for a period of one to two weeks. Subsequently, treatment can be continued with itraconazole 200 mg every eight hours for three days, followed by 200 mg every 24 hours for one year [8]. Furthermore, the 2022 update of the IDSA guidelines recommends the use of voriconazole or posaconazole in cases of central nervous system disease [11]. In the case of the patient presented in this study, he responded adequately to treatment with amphotericin B deoxycholate and was subsequently de-escalated to itraconazole. Despite not having immunosuppression secondary to HIV infection, other causes or conditions that may alter the immune system must be ruled out in the diagnostic approach to disseminated histoplasmosis.

## Conclusions

Mexico is an endemic country for *Histoplasma capsulatum* infection. Therefore, when diagnosing an acute respiratory infection, it is crucial to consider this microorganism as a differential diagnosis, particularly in light of the patient's risk factors and clinical history. In addition to this, the management of disseminated histoplasmosis must be multidisciplinary, as it necessitates close monitoring of the patient's respiratory, metabolic, and immunological functions. Furthermore, continuing education for health professionals and patients enhances the quality of care provided. Additionally, avoiding areas with high nitrogen content or administering prophylactic management in immunosuppressed patients can help prevent the development of this disease.
